# A cross-sectional study of pulmonary and quality of life outcomes in patients with CABG and COPD

**DOI:** 10.3389/fmed.2025.1696441

**Published:** 2026-01-08

**Authors:** Ozlem Cakirkose, Ruhsel Corut, Ali Muhtaroglu, Ersin Kuloglu, Berkan Acar

**Affiliations:** 1 Giresun Faculty of Medicine, Department of Cardiovascular Surgery, Giresun University, Giresun, Türkiye; 2 Giresun Faculty of Medicine, Department of Chest Diseases, Giresun University, Giresun, Türkiye; 3 Giresun Faculty of Medicine, Department of General Surgery, Giresun University, Giresun, Türkiye; 4 Giresun Faculty of Medicine, Department of Internal Medicine, Giresun University, Giresun, Türkiye

**Keywords:** chronic obstructive pulmonary disease, coronary artery bypass grafting, pulmonary function tests, respiratory quality of life, St. George’s Respiratory Questionnaire

## Abstract

**Background:**

Pulmonary dysfunction and compromised respiratory quality of life are frequent sequelae in both coronary artery bypass grafting (CABG) patients and those with chronic obstructive pulmonary disease (COPD). In this study, we compared pulmonary function and respiratory-related quality of life between these two patient groups based on real-life clinical data.

**Methods:**

A total of 89 patients (42 CABG and 47 COPD) were assessed. Baseline demographics, body mass index (BMI), pulmonary function tests (FVC, FEV1, and FEV1/FVC), and St. George’s Respiratory Questionnaire (SGRQ) scores were compared. Comparisons between groups were made using *t*-tests and chi-squared tests. Correlation and multiple linear regression analyses identified determinants of FEV1 (%) and SGRQ total scores.

**Results:**

The groups were comparable in age and sex, but BMI was higher in the CABG group (*p* = 0.002). CABG patients demonstrated significantly better SGRQ total, symptom, and activity scores than COPD patients (*p* < 0.05). Pulmonary function was also superior in the CABG group for FVC (%), FEV1 (%), and FEV1/FVC (all *p* < 0.05). Correlation analyses revealed strong associations between lower SGRQ scores and better pulmonary function. A multiple regression analysis identified “group” (CABG vs. COPD) as the only significant predictor of both FEV1 (%) (*β* = −0.25, *p* = 0.032) and SGRQ total score (*β* = 0.29, *p* = 0.016).

**Conclusion:**

Patients with prior CABG exhibit more favorable lung function and respiratory quality of life than COPD patients. Group status was independently associated with outcomes, suggesting that both groups need tailored rehabilitation and multidisciplinary management. Therefore, multicenter prospective studies are warranted.

## Introduction

Coronary artery bypass grafting (CABG) and chronic obstructive pulmonary disease (COPD) are both common conditions with significant morbidity and compromised respiratory health globally. COPD is a major cause of disability and death, with persistent airflow limitation and progressive pulmonary function decline that can significantly affect daily life and quality of life. Meanwhile, CABG continues to be a mainstay treatment for severe coronary artery disease, but the perioperative course frequently entails pulmonary complications with a potential additive risk of long-term respiratory dysfunction in such patients (1–3).

The impact of COPD goes beyond lung function impairment to also encompass significant functional disability, decreased exercise capacity, and a significant worsening of health-related quality of life (HRQoL) (4, 5). Pulmonary post-surgical complications are also common in cardiac surgery patients, which further increases the risk of chronic respiratory dysfunction. Specifically, dyspnea, fatigue, and restricted physical activity continue in both groups and are robust predictors of overall prognosis as well as daily function (6–8).

Although numerous studies have assessed pulmonary function and quality of life after cardiac or pulmonary disease, there remains a paucity of direct comparative data between post-CABG and COPD patients using standardized, validated patient-reported outcomes. The St. George’s Respiratory Questionnaire (SGRQ) is a widely recognized instrument for evaluating disease impact on daily life in chronic respiratory diseases, and its use in both populations enables more objective, patient-centered comparisons (9–11).

Existing studies often examine these populations separately. However, patients with COPD and prior CABG often exhibit overlapping profiles in clinical practice regarding respiratory symptoms (dyspnea and exercise intolerance) and quality of life limitations. Despite this similar clinical presentation, their underlying pathophysiology is fundamentally different: COPD involves irreversible, progressive airway obstruction, while post-CABG respiratory dysfunction usually represents a transient or partially reversible condition caused by the acute stress of surgery, thoracotomy, and sedation. Therefore, directly comparing these two groups with standardized measures is valuable to understand not only the magnitude of outcomes but also the qualitatively different effects on respiratory health of recovery from an acute physiologic stressor vs. a chronic progressive disease. Such a comparison can guide expectations for both groups, assess prognosis, and ultimately develop targeted rehabilitation and management strategies that address different recovery potentials and pathophysiologic needs.

## Materials and methods

The protocol of the study was approved by the local ethics committee of our hospital on 23 December 2023 (Decision No: 25.12.2023/35). The study was carried out in compliance with the principles of the Declaration of Helsinki, as revised in 2013. Written informed consent was obtained from all participants. This was a cross-sectional study carried out in a single tertiary health center for 1 year. A total of 89 patients aged 18 years and older were enrolled, including 42 patients who had previously undergone CABG and 47 patients with COPD. The patients were recruited consecutively from cardiothoracic surgery and pulmonology outpatient clinics to obtain a representative sample for both groups. The inclusion criteria were a documented history of CABG at least 3 months before enrollment, or a physician-confirmed diagnosis of COPD based on spirometric criteria (FEV1/FVC < 0.70). Patients were excluded if they had ongoing infections, uncontrolled comorbid conditions such as severe heart failure or advanced renal impairment, or other acute illnesses that could potentially have a significant impact on pulmonary function measurements (e.g., recent pneumonia or acute COPD exacerbation).

Demographic information (age, sex, and body mass index [BMI]) and pertinent medical histories were gathered through patient interviews and examination of medical records. Pulmonary function tests were conducted by technicians trained in spirometry using a calibrated spirometer (NDD Medical Technologies AG, CH-8005 Zürich, Switzerland) following American Thoracic Society guidelines. Measured variables were forced vital capacity (FVC, liters and % predicted), forced expiratory volume in 1 s (FEV1, liters and % predicted), and FEV1/FVC ratio (percentage). The patients were asked to avoid smoking, taking large meals, or using short-acting bronchodilators for at least 6 h prior to testing. A minimum of three acceptable maneuvers was performed for each patient, and the optimal values were noted.

All patients also completed the St. George’s Respiratory Questionnaire (SGRQ), a validated tool for measuring respiratory quality of life. The SGRQ contains 50 items across three domains: symptoms (8 items), activity (16 items), and impacts (26 items). Each item has an empirically derived weight. The symptoms domain assesses respiratory discomfort, cough, sputum, wheeze, and breathlessness. The activity domain assesses limitations in physical activities due to breathlessness. The impact domain assesses work, social functioning, control over health, panic, medication effects, and discomfort when performing daily activities. Each domain is scored separately, and an overall score is calculated, which ranges from 0 (normal function) to 100 (extreme disability). A four-unit change is considered clinically significant.

All data were analyzed using SPSS version 26.0 (IBM Corp., Armonk, NY, USA). Normality of continuous variables was assessed using skewness and kurtosis (reference range: ±1.96). Descriptive statistics were calculated. Categorical variables were compared with the chi-squared test; continuous variables (SGRQ scores and pulmonary function parameters) were compared using independent samples t-tests. Pearson’s correlation coefficients were calculated to assess associations between clinical and functional variables. A multiple linear regression analysis was performed to identify predictors of FEV1 (%) and SGRQ total score. Statistical significance was set at *p* < 0.05, with *p* < 0.01 considered highly significant.

## Results

A total of 89 patients were examined, including 42 patients in the CABG group and 47 patients in the COPD group. Between-group differences were not significant in the distribution of sex (male: 78.6% vs. 91.5%, *p* = 0.155), mean age (63.53 ± 8.69 vs. 65.55 ± 10.97 years, *p* = 0.371), and height (167.06 ± 7.59 cm vs.169.45 ± 7.80 cm, *p* = 0.180). The percentage of patients aged less than 65 years was comparable between the groups (47.6% vs. 42.6%), as was the percentage of patients aged 65 years and older (52.4% vs. 57.4%; *p* = 0.790). The mean body weight, however, was significantly greater in the CABG group (83.56 ± 12.1 kg) than in the COPD group (76.55 ± 15.41 kg; *p* = 0.032). Mean body mass index (BMI) was also significantly greater in the CABG group (29.98 ± 4.30 kg/m^2^) than in the COPD group (26.63 ± 4.94 kg/m^2^; *p* = 0.002). When BMI was categorized, the frequency of normal weight, overweight, and obesity did not differ significantly between groups (*p* = 0.368), although the proportion of obese individuals was higher in the CABG group (42.9%) than in the COPD group (29.8%) (Table 1).

**Table 1:** Baseline demographic and clinical characteristics of the study groups.

Variable	CABG (*n* = 42)	COPD (*n* = 47)	*p*-value
Sex (Male/Female)	33 (78.6%) / 9 (21.4%)	43 (91.5%) / 4 (8.5%)	0.155
Age, years	63.53 ± 8.69 (40–83)	65.55 ± 10.97 (23–87)	0.371
Age <65, *n* (%)	20 (47.6%)	20 (42.6%)	0.790
Age ≥65, *n* (%)	22 (52.4%)	27 (57.4%)	
Height, cm	167.06 ± 7.59 (150–180)	169.45 ± 7.80 (150–186)	0.180
Weight, kg	83.56 ± 12.1 (55–106)	76.55 ± 15.41 (50–120)	0.032*
BMI, kg/m^2^	29.98 ± 4.30 (22.03–38.67)	26.63 ± 4.94 (19.49–42.52)	0.002**
BMI status			0.368
Normal, *n* (%)	9 (21.4%)	15 (31.9%)	
Overweight, *n* (%)	15 (35.7%)	18 (38.3%)	
Obese, *n* (%)	18 (42.9%)	14 (29.8%)	

Data are presented as mean ± standard deviation (min–max) or *n* (%).

*p < 0.05, **p < 0.01; *p*-values calculated by the chi-squared test (categorical variables) or the independent samples *t*-test (continuous variables).

CABG, coronary artery bypass grafting; COPD, chronic obstructive pulmonary disease; BMI, body mass index.

Health-related respiratory quality of life was assessed by the SGRQ. The CABG group had significantly more favorable scores in several SGRQ domains than the COPD group. In particular, the SGRQ total score was significantly lower in the CABG group (38.97 ± 20.20) than in the COPD group (49.31 ± 20.57; *p* = 0.019), indicating better respiratory quality of life. When the individual domains were examined, the CABG group had a significantly lower symptom score (38.13 ± 20.15 vs. 51.76 ± 23.39; *p* = 0.004) and activity score (49.02 ± 25.51 vs. 60.80 ± 25.67; *p* = 0.033) than the COPD group, suggesting fewer respiratory symptoms and less impairment in physical activity. However, the difference in impact scores between the two groups did not reach statistical significance (29.79 ± 23.59 vs. 35.35 ± 19.79; *p* = 0.230). These findings indicate that, overall, patients who had undergone CABG reported significantly better respiratory quality of life and fewer disease-related symptoms and activity restrictions than patients with COPD (Table 2).

**Table 2:** Comparison of St. George’s Respiratory Questionnaire (SGRQ) scores between groups.

SGRQ domain	CABG (*n* = 42)	COPD (*n* = 47)	*p*-value
Total score	38.97 ± 20.20	49.31 ± 20.57	0.019*
Symptom score	38.13 ± 20.15	51.76 ± 23.39	0.004**
Activity score	49.02 ± 25.51	60.80 ± 25.67	0.033*
Impact score	29.79 ± 23.59	35.35 ± 19.79	0.230

Data are presented as mean ± standard deviation. SGRQ, St. George’s Respiratory Questionnaire.

*p < 0.05, **p < 0.01; *p*-values calculated by the independent samples *t*-test.

CABG, coronary artery bypass grafting; COPD, chronic obstructive pulmonary disease.

Pulmonary function test results for both groups are shown in Table 3. The FVC (%) was significantly higher in CABG patients (66.32 ± 14.03) than in COPD patients (58.87 ± 17.39), with a mean difference of 7.45% (95% CI: 0.45–14.45; *p* = 0.037). Similarly, the FEV1 (%) was significantly greater in the CABG group (73.22 ± 17.07) than in the COPD group (60.32 ± 21.41), with a mean difference of 12.90% (95% CI: 4.18–21.62; *p* = 0.004). Furthermore, CABG patients had a much better FEV1/FVC ratio (109.54 ± 12.2% vs. 101.93 ± 15.08%), with a mean difference of 7.61% (95% CI: 1.47–13.75; *p* = 0.016) (Table 3).

**Table 3:** Comparison of the pulmonary function test results between the groups.

Parameter	CABG (*n* = 42)	COPD (*n* = 47)	Mean difference (CABG – COPD)	95% CI for difference	*p*-value
FVC (%)	66.32 ± 14.03	58.87 ± 17.39	7.45	0.45–14.45	0.037*
FEV1 (%)	73.22 ± 17.07	60.32 ± 21.41	12.90	4.18–21.62	0.004**
FEV1/FVC (%)	109.54 ± 12.2	101.93 ± 15.08	7.61	1.47–13.75	0.016*

Data are presented as mean ± standard deviation. FVC, forced vital capacity; FEV1, forced expiratory volume in 1 s.

*p < 0.05, **p < 0.01; *p*-values calculated by the independent samples *t*-test.

CABG, coronary artery bypass grafting; COPD, chronic obstructive pulmonary disease.

In CABG patients, BMI showed positive correlations with FVC% (*r* = 0.228, *p* = 0.037), FEV1% (*r* = 0.314, *p* = 0.004), and SGRQ total score (*r* = 0.343, *p* = 0.002), whereas these correlations were inverse in the COPD group (*r* = −0.228, *p* = 0.037; *r* = −0.314, *p* = 0.004; *r* = −0.343, *p* = 0.002, respectively). SGRQ total score correlated negatively with FVC% (*r* = −0.248, *p* = 0.019) and FEV1% (*r* = −0.314, *p* = 0.004) in the CABG group, while the same relationships were positive in COPD patients (FVC%: *r* = 0.248, *p* = 0.019; FEV1%: *r* = 0.314, *p* = 0.004). Furthermore, there was a positive correlation (*r* = 0.314, *p* = 0.004) between FEV1% and SGRQ total score among patients in the COPD group, but an inverse correlation (*r* = −0.314, *p* = 0.004) between FEV1% and SGRQ total score among patients in the CABG group (Table 4).

**Table 4:** Correlation coefficients between SGRQ scores, pulmonary function test results, and demographic/clinical parameters in the CABG and COPD groups.

Variable	CABG (*n* = 42)		COPD (*n* = 47)	
	R	*p*-value	*r*	*p*-value
BMI with:				
FVC (%)	0.228	0.037*	−0.228	0.037*
FEV1 (%)	0.314**	0.004**	−0.314	0.004**
SGRQ total score	0.343	0.002**	−0.343	0.002**
SGRQ total with:				
FVC (%)	−0.248	0.019*	0.248	0.019*
FEV1 (%)	−0.314	0.004**	0.314	0.004**
FEV1 (%) with:				
SGRQ total score	−0.314	0.004**	0.314	0.004**

Data are presented as the Pearson’s correlation coefficient (*r*) and *p*-value for each group (CABG and COPD). *p < 0.05, **p < 0.01.

CABG, coronary artery bypass grafting; COPD, chronic obstructive pulmonary disease; BMI, body mass index; SGRQ, St. George’s Respiratory Questionnaire; FVC, forced vital capacity; FEV1, forced expiratory volume in 1 s.

A multiple linear regression analysis was conducted to identify independent predictors of pulmonary function (FEV1%) and respiratory quality of life (SGRQ total score) (Table 5). For FEV1 (%), age, sex, and BMI did not reach statistical significance as predictors (all *p* > 0.05), although there was a trend toward a negative association with age (B = −0.44, *p* = 0.053) and a positive association with BMI (B = 0.84, *p* = 0.087). The only significant independent predictor was patient group: assignment to the COPD group (vs. CABG) was associated with a significantly lower FEV1 (%) (B = −10.07, *β* = −0.25, *p* = 0.032), with the model explaining 21.0% of the variance (R^2^ = 0.210). In the model predicting SGRQ total score, age, sex, and BMI again were not significant (*p* > 0.05), but group status remained significant: patients with COPD had higher SGRQ total scores, indicating worse respiratory quality of life (B = 12.37, *β* = 0.29, *p* = 0.016). The explanatory power of this model was lower (R^2^ = 0.065). These results underscore that, after adjusting for demographic factors and BMI, the diagnostic group (CABG vs. COPD) is the principal determinant of both pulmonary function and health-related respiratory quality of life in this population (Table 5).

**Table 5:** Multiple linear regression analysis of factors associated with FEV1 (%) and SGRQ total score.

Dependent variable	Independent variable	B	SE	*β* (Beta)	95% CI for difference	*p*-value	R^2^
FEV1 (%)	Age (years)	−0.44	0.22	−0.21		0.053	
FEV1 (%)	Sex (male = 0, female = 1)	−7.66	6.30	−0.13		0.228	
FEV1 (%)	BMI (kg/m^2^)	0.84	0.49	0.20		0.087	
FEV1 (%)	Group (CABG = 0, COPD = 1)	−10.07	4.60	−0.25	(−19.20 to −0.94)	0.032*	0.210
SGRQ total score	Age (years)	0.22	0.24	0.10		0.367	
SGRQ total score	Sex (male = 0, female = 1)	7.72	6.87	0.13	(2.40 to 22.34)	0.265	
SGRQ total score	BMI (kg/m^2^)	0.87	0.53	0.20		0.105	
SGRQ total score	Group (CABG = 0, COPD = 1)	12.37	5.02	0.29		0.016*	0.065

B, Unstandardized coefficient; SE, standard error; β (beta), standardized coefficient; R^2^, proportion of variance explained by the model.

Sex coded as male = 0, female = 1; group coded as CABG = 0, COPD = 1.

*p < 0.05.

FEV1, forced expiratory volume in 1 s; SGRQ, St. George’s Respiratory Questionnaire; BMI, body mass index; CABG, coronary artery bypass grafting; COPD, chronic obstructive pulmonary disease.

## Discussion

This research provides a direct and comprehensive comparison of pulmonary function and respiratory-related quality of life between subjects with COPD and those with CABG-related features. A major strength of our study is its direct comparison of two populations that are clinically similar but pathophysiologically distinct. The better spirometric values and SGRQ scores observed in the CABG group likely reflect the relatively reversible nature of post-surgical lung dysfunction, which tends to improve over time, compared to the chronic, degenerative nature of COPD. This finding points to an important implication for clinical practice: in patients presenting with similar respiratory complaints, the underlying diagnosis (chronic COPD vs. post-surgical recovery) determines prognosis and the potential for functional recovery. Consequently, while the focus for post-CABG patients may be on time-limited, intensive rehabilitation programs aimed at restoring functional capacity, a continuous, comprehensive management model focused on symptom control and slowing disease progression will be necessary for patients with COPD.

Consistent with our findings, previous research has demonstrated that patients generally achieve significant improvements in pulmonary function and respiratory quality of life following CABG, despite the initial risk of perioperative pulmonary complications (12, 13). Long-term follow-up studies confirm that gains in FVC, FEV1, and patient-reported health status are often sustained for months or years after surgery, highlighting the potential for meaningful recovery in this patient group. In contrast, COPD is clearly a progressive disease characterized by chronic airflow limitation, worsening symptoms, and a significant decline in health-related quality of life. Previous studies have consistently shown that chronic airflow obstruction, dyspnea, and reduced exercise tolerance in COPD contribute to significant functional impairment and disability (14, 15). The SGRQ is widely accepted for the measurement of clinically important differences in respiratory symptoms and impairments in chronic pulmonary diseases (11, 12). The significant differences in SGRQ total, symptom, and activity score observed in our research are consistent with previous research and support the use of this measurement tool in differentiating between patient populations with different disease courses for the tool (12, 16, 17). In short, these findings concur with the chronic and disabling nature of COPD, with the frequently reversible and surgical-related impairments that can occur following CABG.

These results are therefore helpful for clinical practice and multimodal patient care. The finding that post-CABG subjects had better pulmonary function and respiratory quality of life than post-COPD subjects indicates the need for specific rehabilitation protocols for the two populations. In clinical practice, patients recovering from CABG may benefit from structured cardiopulmonary rehabilitation programs that support lung function recovery and help maintain functional capacity, as demonstrated in several controlled studies (2, 15). In contrast, individuals with COPD—who experience progressive and persistent symptoms—require comprehensive, ongoing pulmonary rehabilitation and symptom management strategies to preserve daily functioning and quality of life (5, 18). By combining standardized patient-generated measures such as the SGRQ with objective spirometric assessments, further monitoring is made possible for disease progression, and the effect of treatment and follow-up at the longer term is tailored (10, 11, 16, 17). Another significant remark is that these multidimensional instruments offer clinically actionable information for decision-making during pulmonological and cardiological practice. Our data advocate for the use of multidisciplinary care and repeated quality-of-life assessments as part of standard follow-up for both populations, particularly to enable repeated follow-up specifically for the purpose of earlier detection and care for subjects with a higher risk of functional decline.

This strong interrelation between objective measures of pulmonary function and subjective quality of life thus further supports the utility of multidimensional assessment for research and clinical purposes. Correlation calculations in our study reaffirmed that lower SGRQ total, symptom, and activity scores were associated with preserved pulmonary function, namely FEV1 (%), for the two patient populations. These results concur with earlier reports detailing the strong interrelation between spirometric indices and health status for chronic respiratory disease (16, 17, 19, 20). In our multivariate regression analysis, conventional demographic factors such as age, sex, and BMI were not independently predictive of FEV1 (%) or SGRQ total score when controlling for group status. This further emphasizes the overwhelming effect of disease category—CABG recovery compared with COPD progression—on long-term outcome, a relationship also observed in multicenter clinical research (13, 21, 22). The variable relationship of BMI between the two populations, with higher BMI in concordance with more preserved pulmonary function for CABG subjects but increased deficit in COPD subjects, is in accordance with earlier reports detailing an interactive relationship between body composition and disease course in complex terms (6, 23, 24). In short, these results indicated that group assignment is the most significant determinant of pulmonary function and respiratory-related quality of life, underscoring the importance of care strategies specifically designed for the individual.

The divergent relationship between BMI and outcomes in the two groups is noteworthy. In CABG patients, a higher BMI was correlated with better pulmonary function (FEV1%) and quality of life (SGRQ), whereas in COPD patients, the opposite relationship was observed. This finding aligns with the concept of the ‘obesity paradox,’ a phenomenon documented in cardiovascular populations, including post-cardiac surgery patients, where overweight and mild obesity have been associated with lower mortality and potentially better functional recovery (25, 26). Potential mechanisms for this phenomenon in CABG patients may include greater metabolic reserves for coping with surgical stress, a potential anti-inflammatory role for adipokines, or the confounding effect of more severe disease leading to cardiac cachexia in lower-BMI individuals (27). In contrast, for COPD patients, a higher BMI is often a component of the metabolic syndrome and can exacerbate the mechanical load on already compromised respiratory muscles, worsen gas exchange, and contribute to systemic inflammation, thereby leading to poorer pulmonary function and quality of life (28, 29). While our cross-sectional data cannot prove causality, this contrasting pattern underscores the distinct pathophysiological landscapes of these diseases. It suggests that the prognostic implications of BMI are context-dependent and reinforces the need for disease-specific management strategies that consider body composition.

Our findings demonstrate superior pulmonary function and respiratory-related quality of life in post-CABG patients than in COPD patients. This finding is, however, largely anticipated given the fundamental differences in the underlying pathophysiology and chronicity of the two conditions. COPD is a primary, progressive pulmonary disease characterized by irreversible airflow limitation and persistent inflammation. In contrast, the pulmonary impairment in CABG patients is typically secondary, often stemming from perioperative factors such as anesthesia, sternotomy, and cardiopulmonary bypass, and may be partially or fully reversible with time and appropriate rehabilitation (2, 10, 15). Therefore, the superior spirometric and SGRQ values observed in the CABG group reflect these distinct disease trajectories. While our study robustly identifies ‘group status’ as the strongest predictor of outcomes within this cohort, the cross-sectional design and potential for unmeasured confounders, such as detailed cardiac function, the severity of coronary artery disease, or specific perioperative data in the CABG group, preclude definitive causal inferences. Consequently, these results should not directly dictate changes in clinical practice for either group. Instead, they serve to generate important hypotheses and underscore the necessity for larger, multicenter, prospective studies that incorporate more detailed phenotyping. Such future research is essential to validate these findings, elucidate the long-term trajectory of pulmonary outcomes in CABG patients, and ultimately guide the development of tailored, multidisciplinary rehabilitation strategies for these distinct patient populations.

Despite its contributions, this study has several limitations that should be considered when interpreting the findings. The cross-sectional design precludes the assessment of causality or the longitudinal trajectory of pulmonary recovery post-CABG or progression of COPD. Our sample size, while adequate for initial group comparisons, may limit the statistical power for more complex subgroup analyses or for detecting smaller effect sizes among covariates, and the single-center recruitment may affect the generalizability of our results to other healthcare settings or populations.

Furthermore, several potential confounding variables were not accounted for in our analysis. Several potential confounding variables were not measured or adjusted for in our analysis. While the groups were comparable in terms of age and sex, other factors known to influence pulmonary function and quality of life—such as detailed smoking history (pack-years, current status), the precise time elapsed since CABG surgery, medication use (e.g., bronchodilators and corticosteroids), baseline cardiac function (e.g., ejection fraction), and specific comorbidities—were not accounted for. The absence of this data indicates that residual confounding is a possibility, and the observed differences, although stark, cannot be solely and definitively attributed to the group designation (CABG vs. COPD). For instance, a longer time since surgery in the CABG group could favor better recovery, and differences in smoking prevalence could independently impact outcomes. While our consecutive sampling from a real-world clinical setting enhances external validity, it also introduces the potential for selection bias, as patients attending our tertiary clinic may not be fully representative of the broader population. Therefore, the identified association between patient group and outcomes should be interpreted as strong and clinically informative, but causal inference is limited by these unmeasured confounders. Detailed smoking history (pack-years, current vs. former status), the precise time elapsed since CABG surgery, COPD severity stratification (e.g., GOLD stage), preoperative pulmonary optimization, and specific medication use (such as bronchodilators or inhaled corticosteroids) could significantly influence both pulmonary function and quality of life. Their absence from our models indicates that residual confounding is a possibility. For instance, the significantly higher BMI in the CABG group, while adjusted for in regression, may represent a complex confounding factor rather than a simple demographic difference, potentially mediating some of the observed group differences in outcomes.

Finally, the sample size of this study (*n* = 89) was modest and was not based on a pre-specified power analysis. While the consecutive sampling method enhances representativeness within our center, the sample may be underpowered to detect smaller, yet potentially clinically relevant, effect sizes or to conduct robust subgroup analyses. The moderate explanatory power (R^2^) of our regression models, especially for the SGRQ outcome, further suggests that other unmeasured factors play a significant role. Therefore, our findings, particularly the null associations for covariates such as age and BMI in the regression models, should be interpreted with caution. The results are best viewed as generating reliable evidence for the large effect of group assignment in this initial comparison, while future studies with larger, prospectively determined sample sizes are needed to confirm these relationships and explore more nuanced predictors.

## Conclusion

In the future, larger, multicenter, prospective cohort studies are warranted to validate and extend our findings. Such studies should aim to recruit larger samples to enhance statistical power for subgroup analyses and adjust for potential confounders that could not be fully addressed in our study. These include detailed smoking history (pack-years, cessation status), COPD severity stratification (e.g., GOLD stage), the precise time elapsed since CABG surgery, medication regimens (e.g., bronchodilators and corticosteroids), and comprehensive comorbidity profiles. Incorporating longitudinal follow-up would be crucial to delineate the recovery trajectories of pulmonary function and quality of life post-CABG and to compare them directly with the natural progression of COPD. This would allow for the assessment of long-term complication rates and responses to rehabilitation in both groups. To minimize residual confounding, future analyses could use propensity score matching or stratified analyses to better account for baseline differences such as BMI. Furthermore, standardizing the timing of spirometry and SGRQ administration in relation to surgery or clinical stability would improve the comparability of results. Ultimately, such rigorous research designs will enhance our understanding of the distinct pathophysiological pathways and care needs in these populations, guiding the development of more precise, effective, and personalized rehabilitation and management strategies.

## Data Availability

The original contributions presented in the study are included in the article/supplementary material, further inquiries can be directed to the corresponding author.
